# Effects of Intra- and Interspecific Plant Density on Rhizosphere Bacterial Communities

**DOI:** 10.3389/fmicb.2020.01045

**Published:** 2020-05-26

**Authors:** Andrea Cavalieri, Frederik Bak, Adriana M. Garcia-Lemos, Jacob Weiner, Mette Haubjerg Nicolaisen, Ole Nybroe

**Affiliations:** Department of Plant and Environmental Sciences, University of Copenhagen, Frederiksberg, Denmark

**Keywords:** *Centaurea cyanus*, *Dracocephalum moldavica*, *Lolium multiflorum*, *Plantago psyllium*, *Trifolium repens*, nitrogen-fixing bacteria, plant density, root microbiome

## Abstract

There have been very few studies on the effects of plant competition on the rhizosphere bacterial community. To investigate the impacts of intra- and interspecific plant competition, we analyzed the responses of rhizosphere bacterial communities to plant density as determined by 16S rRNA gene targeted sequencing. We included five weedy plant species growing in field soil in monocultures and mixed cultures at three densities in a greenhouse experiment. The rhizosphere bacterial community of each species changed more with density in a mixture of all five plant species than in monocultures, so intra- and interspecific plant competition had different effects on the bacterial community. For the dominant plant competitor, *Centaurea cyanus*, neither intra- nor interspecific competition had major effects on the composition of its rhizosphere bacterial communities. In contrast, the bacterial communities of the weakest competitor, *Trifolium repens*, were affected differently by intra- and interspecific competition. During increasing intraspecific competition *T. repens* maintained a highly specialized bacterial community dominated by *Rhizobium*; while during interspecific competition, the relative abundance of *Rhizobium* declined while other nitrogen fixing and potentially plant growth promoting taxa became more abundant. Contrary to previous observations made for soil microbial communities, the bacterial rhizosphere community of the weakest competitor did not become more similar to that of the dominant species. Thus, the process of competition, as well as the plant species themselves, determined the rhizosphere bacterial community. Our results emphasize the role of plant-plant interactions for rhizosphere bacterial communities. These effects may feedback to affect plant-plant interactions, and this is an important hypothesis for future research.

## Introduction

Plant roots are colonized by complex microbial communities that may reside in the rhizosphere soil or live as root-attached or endophytic communities in closer association with the roots (Philippot et al., 2012; Mendes et al., 2013). The rhizosphere microbiota is primarily recruited from the surrounding soil, and the soil type as well as the plant genotype represent two important factors shaping the composition of rhizosphere microbial communities (Vandenkoornhuyse et al., 2015; Jacoby et al., 2017).

Root-associated microbial communities contain beneficial microorganisms as well as plant pathogens, among which fungi and oomycetes represent the most important soil-borne groups (Prashar et al., 2014; Ciancio et al., 2016). Beneficial microorganisms may protect plants from these pathogenic microorganisms (Mendes et al., 2013). Furthermore, rhizosphere microbiota can play important roles by increasing the availability of nutrients for the plants (Lugtenberg and Kamilova, 2009; Guyonnet et al., 2018). For example, plants depend on microorganisms for depolymerizing, solubilizing and mineralizing nitrogen, phosphorous, and sulfur from sources in the soil (Jacoby et al., 2017). Additionally, fixation of atmospheric nitrogen performed by diazotrophs is particularly important for ammonium acquisition by legumes (Richardson et al., 2009), although non-leguminous plants have also been found to host nitrogen-fixing bacteria (Santi et al., 2013).

Analyses of rhizosphere microbiota have predominantly been performed on individual plants. Plants rarely live alone, however, but interact with neighboring plants in the field. Plant-plant interactions can be both positive (facilitation) and negative (competition). In most plant communities, the latter dominate, reducing individual plant growth, including root growth, and although positive effects can also be important in some situations (Gallandt and Weiner, 2015), competition among plants is one of the major determinants of plant community structure and dynamics (Hortal et al., 2017). Neighboring plants can belong to the same or different species, referred to as intra- and interspecific interactions, respectively. According to basic principles of community ecology, stable coexistence of different species will occur if intraspecific competition is stronger than interspecific competition (Begon et al., 2005).

Plants respond to the presence of neighboring plants through their growth rate and growth form, and they also respond physiologically and biochemically, e.g., by changes in root exudate composition (Broz et al., 2010; Badri et al., 2012; Pierik et al., 2013). These changes may affect the composition of microbial communities in the soil or associated with roots (Badri et al., 2009; Wenke et al., 2010; Chaparro et al., 2013). There is evidence for a microbial component of plant competition (Siefert et al., 2018). Under mixed culture interspecies competition with two plant species, it was recently shown that the soil microbial community collected between plants resembled that of soil samples from monocultures of the superior competitor (Hortal et al., 2017). However, little, if anything, is known about the plant density-dependent effects of intra- versus interspecific competition on the microbiota directly associated with the roots of competing plants. Furthermore, the effect of plant density on rhizosphere microbiota has only been studied in monocultures of canola (Lay et al., 2018). Very few studies have addressed changes in rhizosphere microbial communities in response to interspecific competition, although a recent study showed that phytohormone signaling from interspecific neighbors can affect rhizosphere microbial community assembly (Chen et al., 2020).

In the present study we investigate a model community of five co-occurring weed species from three functional groups from semi-natural and agricultural environments: the grass *Lolium multiflorum* Lam (Italian ryegrass), the legume *Trifolium repens* L (white clover), and three herbaceous dicots: *Dracocephalum moldavica* L (Moldavian dragonhead), *Centaurea cyanus* L (cornflower), and *Plantago psyllium* L (psyllium).

The objectives of the current study were to (1) ask if plant density affects the rhizosphere bacterial community of single species populations, (2) determine the composition of rhizosphere bacterial communities of these plant species in monocultures versus mixed cultures, and (3) clarify the effects of intra- versus interspecific competition on the microbial communities at increasing plant densities. We hypothesized that (a) individual plants maintain a specific microbiome across plant densities and (b) under increasing interspecific competition, the dominant plant species maintain its root microbiome while the root microbiome of the inferior competitor changes, and become more similar to that of the dominant.

## Materials and Methods

### Plant Species

We selected five common, co-occurring plant species from temperate European herbaceous semi-natural and agricultural environments, representing three of the most important functional groups in these communities: one grass, *L. multiflorum*, one legume, *T. repens*, and three herbaceous dicots, *D. moldavica* L., *C. cyanus*, and *P. psyllium*. The plant species chosen are annuals, except for the perennial *T. repens*, which often behaves as an annual. Commercial seeds were purchased from PHARMASAAT Arznei- und Gewürzpflanzensaatzucht GmbH (Artern/Unstrut, Germany), and Sow Seeds Limited (Brough, United Kingdom). Before starting the experiment, germination tests were performed in petri dishes (10 cm Ø), and sowing target densities were calculated accounting for the germination rate. Since the species varied greatly in seed mass, we sowed them in equal proportions of total seed mass, not seed number, at each density, starting with a density of 175 seeds m^–2^ of the species with the largest seeds [*C. cyanus*, thousand-kernel weight (TKW) = 3.98 g] at the lowest density. The density series was logarithmic, corresponding to densities of 175, 612 and 2140 seeds m^–2^ of *C. cyanus*, henceforth referred to as density “Low,” “Medium,” and “High,” respectively. For each density, seeds of *C. cyanus* were counted and weighed, and these weights were then used to adjust the amount of seeds of the other species, both in monoculture and in mixed culture, such that, at each density, the number of seeds differed among species, but the total seed weight and proportions in mixture were the same. Therefore, the weight of seeds of each species was the same at each monoculture density, and the weight of each species in mixture was the same at each mixture density.

### Experimental Design

Experiments were conducted during the spring-summer season 2017 in a greenhouse at the experimental farm of the University of Copenhagen, in Taastrup (55°40’07.2″N, 12°18’19.8″E). Mesocosms (40 liters containers, 45 cm Ø, 30 cm height) were used to correspond as well as possible to field conditions. Soil was collected from the field of the experimental farm. The soil was a sandy-clay (pH 6.9 (CaCl_2_), 12.8 mg P kg^–1^ soil, 4.7 mg K kg^–1^ soil, 2.2 mg Mg kg^–1^ soil). Before filling the containers, commercial potting soil (SW Horto A/S, Bramming, DK) was added (10% of volume) as a source of nutrients and organic matter (pH 5.5-6.5, NPK 14-7-15). The soil was then homogenized with a soil mixer. Plants were grown in monocultures and in five-species mixtures (mixed cultures). Mesocosms were arranged on wood pellets placed on the floor in a randomized complete block design including three replicates for the mixed cultures, while only one replicate was conducted for the monocultures.

Seeds were hand sown by spreading evenly over each mesocosm. The sowing was done in May 2017 (Julian day 125), and the soil watered afterward. The temperature was maintained at 15–20°C, corresponding to the natural summer conditions of the region. When temperatures in the greenhouse were too high, a curtain system was automatically activated to provide shade, but no cooling system was available. No fertilizers or artificial light were used, normal tap water was used for the irrigation and yellow sticky traps were added to monitor pest occurrence.

### Plant Biomass and Molecular Analysis of the Rhizosphere

Plant growth was assessed for both the monocultures and in the five species mixtures by harvesting the total aboveground biomass. All plants were harvested when they had almost reached the physiological maturity (Julian day 185). The aboveground biomass harvested from each mixture was sorted into single species. Plant dry weight was then determined after drying in an oven for 48 h at 60°C.

Plant biomass was used to determine the relative competition intensity (RCI) for each species. RCI compares the performance of plants growing in mixtures with monocultures, using the formula RCI = (B_*mono*_ – B_*mix*_)/B_*mono*_, where B_*mono*_ and B_*mix*_ are the biomass per individual in monoculture and mixture, respectively (Weigelt and Jolliffe, 2003). Negative RCI means that intraspecific competition is stronger than interspecific competition (individuals of the species produced more biomass when growing in mixtures than in pure stands), while a positive RCI indicates that a species experienced stronger interspecific than intraspecific competition (individuals produced less biomass in mixtures than in pure stands).

For the molecular analysis, representative plants of each species were pulled out of the soil, shaken gently to remove superfluous soil, where after roots were collected. Root samples were freeze-dried, grinded and homogenized prior to DNA extraction. DNA was subsequently extracted from the rhizosphere (defined here and used throughout to refer to plant roots with adhering soil) of each of the species, following the protocol of Paulin et al. (2013).

### Sequence Processing

Sequencing libraries were prepared by amplifying the V3–V4 regions of the bacterial 16S rRNA gene using the Bakt 341F primer (5′-CCTACGGGNGGCWGCAG-3′) and the Bakt 805R primer (5′-GACTACHVGGGTATCTAATCC-3′) (Herlemann et al., 2011). Libraries were sequenced on an Illumina MiSeq (Macrogen Inc., Seoul, South Korea) with a read length of 2 × 300 bp. Primers were removed from the raw sequences using cutadapt v. 1.16 (Martin, 2014). Successional processing and quality control was performed in R using the DADA2 package version 1.8.0 following the workflow in the tutorial with few exceptions (Callahan et al., 2016). Reverse reads were truncated to 240 bp and both forward and reverse reads were filtered (maximum of five or seven expected errors per read, respectively). Reads between 375 and 475 bp were kept in the data set. The exact amplicon sequence variants (ASVs) were taxonomically classified with a naïve Bayesian classifier using the Silva v. 132 training set (Pruesse et al., 2007). The ASVs belonging to Archaea, chloroplasts or mitochondria were removed before further analysis. The raw sequences were deposited in NCBI’s Sequence Read Archive under BioProject PRJNA574065.

### Quantitative PCR

The functional gene *nifH* (dinitrogenase reductase) as well as the 16S rRNA gene were quantified using an Mx3000P^®^ qPCR system (Agilent Technologies, Santa Clara, CA, United States). The primers for the 16S rRNA were 341F (CCTAYGGGRBGCASCAG) and 806R (GGACTACNNGGGTATCTAAT) (Yu et al., 2005), while the primer pair for the bacterial *nifH* gene was nifHF (AAAGGYGGWATCGGYAARTCCACCAC) and nifHRb (TGSGCYTTGTCYTCRCGGATBGGCAT) (Zheng et al., 2018). Amplification of each gene was carried out in 20 μl reaction mixtures [10 μl of the Brilliant III Ultra-Fast SYBR^®^ Green Low ROX qPCR Master Mix (Agilent Technologies), 0.015 μM of Bovine Serum Albumin (Thermo Fisher Scientific, Waltham, MA, United States), 0.4 μM of each primer, and 2 μl of template DNA (1 to 10 ng/μl)]. The qPCR thermal cycling consisted of an initial cycle of 95°C for 3 min, followed by 40 cycles of 95°C for 20 s, annealing temperature for 30 s at 58°C for the 16S primers and at 56°C for the *nifH* primers, and a final extension of 1 min at 72°C. Standard curves were constructed as described previously (Garcia-Lemos et al., 2020).

### Statistical Analysis

The analysis of the variance (ANOVA) of the aboveground biomass for the mixed plant communities was performed with mixed models (Sas Institute Inc, 2015) to examine the significance of all factors and their interactions. Fixed factors were plant species and density, while replicates were considered as random effect. Residuals were examined and found for the homogeneity of the distribution.

All analyses of bacterial 16S rRNA sequences were conducted in R. Rarefaction curves were made using custom function ggrare from “phyloseq-extended (Mariadassou, 2019).” Richness and alpha diversity were calculated using the R package “phyloseq” v. 1.24.2 (McMurdie and Holmes, 2013). Community compositions were compared using Bray-Curtis dissimilarities on raw counts and presented using NMDS ordinations. Ordinations and heatmaps were done in the R package “ampvis2” v. 2.4.6 (Andersen et al., 2018). PERMANOVA was calculated using “vegan” v. 2.5-4 (Oksanen et al., 2018). Linear mixed effect models were made with the package “nlme” v. 3.1-137 (Pinheiro et al., 2014) for alpha diversity, richness and qPCR data of the *nifH* genes with plant species, plant stand and density as fixed effects and replicates as random effects to test for normality. The effect of density on the variables was tested using one-way ANOVA. Tests with a *p* < 0.05 were considered significant and were followed by a Tukey HSD *post hoc* test. The effects of monoculture vs. mixture and plant species were tested using a blocked one-way ANOVA with density as the blocking factor.

To visualize the change of bacterial groups in the root microbiomes we grouped ASVs into three groups for each plant: Core, Shared, and Unique. The Core ASVs were found in minimum eight out of the nine microbiomes from a specific plant grown in monoculture and in minimum eight out of nine microbiomes when the plant was grown in the mixed cultures. The Shared group consisted of ASVs identified in the plant rhizosphere microbiomes of both *T. repens* and *C. cyanus* irrespective of relative proportions. However, if an ASV belonged to the Core group it was removed from the Shared group. The Unique group contained ASVs found only in the specific plant (i.e., *T. repens* or *C. cyanus*), excluding the Core ASVs. Hence, no ASVs could belong to two groups. The relative proportions of the three groups were calculated and visualized using “treemapify” v. 2.5.3 (Wilkins, 2019). Visualization of the data was done using the package “ggplot2” (Wickham, 2016).

## Results

### Plant Biomass and Relative Competitive Intensity

**FIGURE 1:**
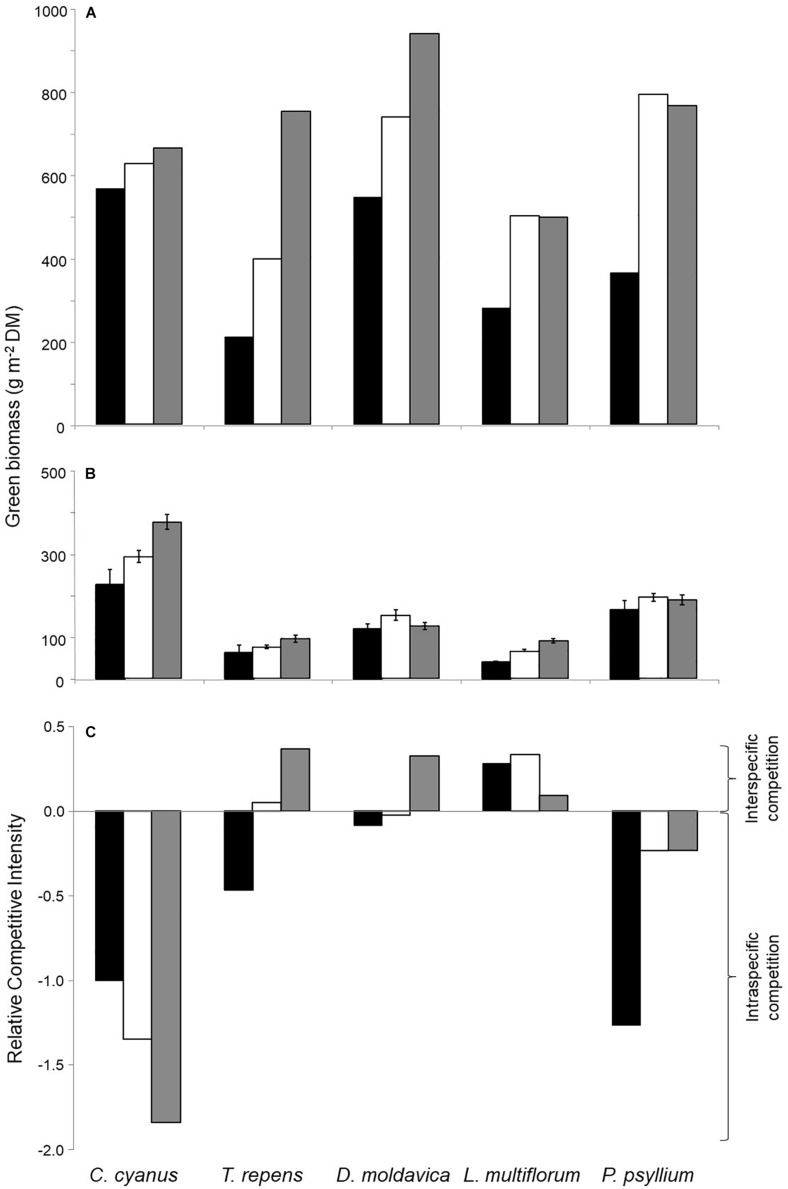
Aboveground biomass of the five plant species at three densities in **(A)** monocultures and **(B)** mixed culture. **(C)** Relative competitive intensity (RCI) of the five species growing at the three densities. Negative RCI represents that species experience stronger intraspecific than interspecific competition (more biomass produced when growing in mixed cultures than in monocultures), while a positive RCI indicates that species experience stronger interspecific than intraspecific competition (produces less biomass growing in mixed cultures than in monocultures. Black bars, low density; white, medium density; gray, high density. Error bars represent standard errors (SE) (*n* = 3).

All plants grown in monocultures showed a higher aboveground biomass at the highest plant density compared with the lowest (Figure 1A). Biomass of *C. cyanus*, *T. repens*, and *D. moldavica* increased with increasing density, while the biomass of *L. multiflorum* and *P. psyllium* leveled off between the medium and the high density. *T. repens* showed the greatest increase in biomass with increasing plant density in monoculture. In the five-species mixed cultures, only *C. cyanus* and *L. multiflorum* showed significantly increasing biomass production with increasing plant density (Figure 1B).

Biomass data from the monocultures and mixed cultures were used to calculate the relative competitive intensity (RCI). The results consistently showed a negative RCI for *C. cyanus* and *P. psyllium* at all densities, reflecting stronger intra- than interspecific competition (Figure 1C). *L. multiflorum* displayed consistent positive RCI values, reflecting stronger effects of interspecific competition. Finally, the RCI of *T. repens* and *D. moldavica* changed from negative values at the lowest plant density to positive values at the highest density indicating that they were increasingly affected by interspecific competition as plant density increased. This was particularly pronounced for *T. repens* (Figure 1C). Thus, *C. cyanus* was the strongest competitor under these experimental conditions, while *T. repens* was the weakest.

### Impact of Plant Species and Density on Rhizosphere Bacterial Communities

**FIGURE 2:**
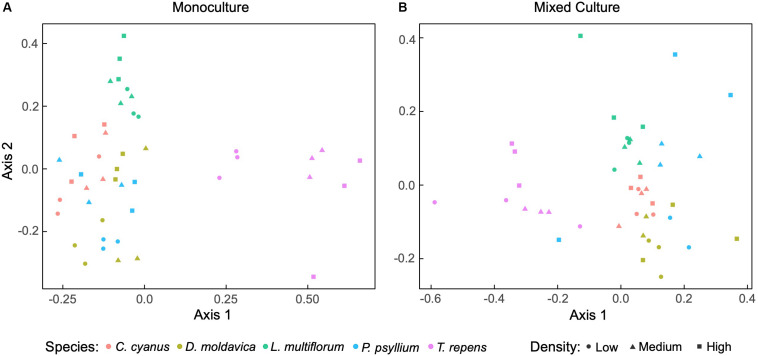
Non-metric multidimensional scaling (NMDS) ordination based on Bray-Curtis dissimilarities of relative abundances of bacterial amplicon sequence variants (ASVs) associated with roots from plants grown in monoculture **(A)** (stress = 0.15) and mixed cultures **(B)** (stress = 0.14) at three plant densities.

We sequenced the V3-V4 regions of the 16S rRNA gene and obtained a total of 1,677,114 sequences from rhizosphere and soil samples after filtering. The rarefaction curves implied that sequencing coverage was sufficient as plateaus were reached for both soil and plant communities (Supplementary Figure S1). For plants grown in monoculture, non-metric multidimensional scaling (NMDS) ordination revealed that the rhizosphere bacterial communities of *T. repens* and *L. multiflorum* clustered separately, while those of *C. cyanus*, *D. moldavica* and *P. psyllium* overlapped extensively (Figure 2A). The analysis also indicated some change in community structure with increasing plant density, most notably for *T. repens*. PERMANOVA results indicated that plant species explained almost half of the variation between communities (R^2^ = 0.55, *p* < 0.001). Plant density and interaction between species and density were not significant (*p* = 0.16 and *p* = 0.06, respectively). NMDS ordination of rhizosphere bacterial communities from the mixed plant cultures showed a distinct separation among the plant species at all densities, although data for *P. psyllium* were highly variable (Figure 2B). PERMANOVA results showed that both plant species (*p* < 0.001) and density (*p* = 0.048) significantly affected the community compositions, while the interaction was not significant (*p* = 0.39). The rhizosphere bacterial communities were dominated by rhizosphere taxa belonging to the phyla Proteobacteria, Actinobacteria, and Firmicutes (Supplementary Figures S2, S3).

### Diversity and Community Composition in Monoculture

**FIGURE 3:**
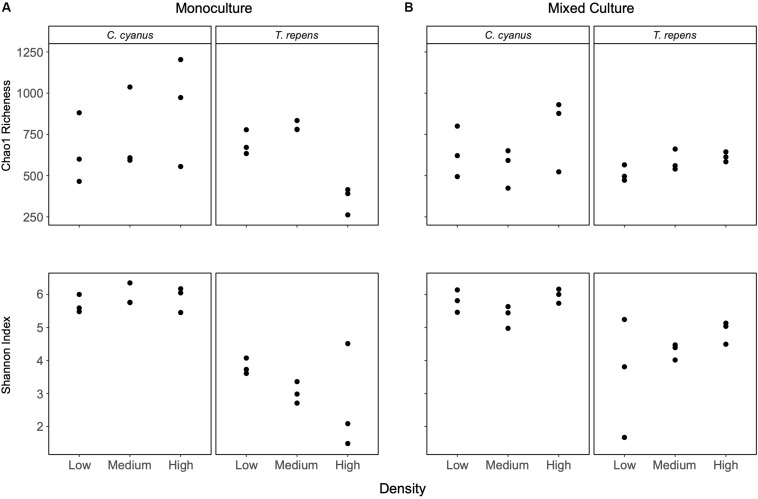
Chao1 richness and alpha diversity (Shannon index) for rhizosphere bacterial communities of *C. cyanus* and *T. repens* grown in monoculture **(A)** or in mixed culture **(B)** at three plant densities (low, medium, and high).

*Centaurea cyanus* was the strongest and *T. repens* the weakest competitor under the experimental conditions (Figure 1), so we focus on the rhizosphere bacterial communities of these two species. Analyses of bacterial communities from the *C. cyanus* monocultures showed that neither the Chao1 richness nor the Shannon index differed between plant densities (*p* = 0.52 and 0.63, respectively; Figure 3A). The Shannon index of the rhizosphere communities of *C. cyanus* was significantly higher than that of *T. repens* communities at all plant densities (*p* < 0.001).

For *T. repens* monocultures, the Chao1 richness of the rhizosphere bacterial communities was significantly lower at the highest plant density compared to the low and medium densities (*p* = 0.0019 and *p* = 0.00046, respectively; Figure 3A). There was no significant effect of plant density on the Shannon index of the communities (see Supplementary Figures S4, S5 for the results for the other species).

The composition of the rhizosphere communities changed when the densities of the plants were increased in monoculture (Supplementary Figure S2). In *C. cyanus* communities, *Streptomyces* increased in relative abundance with density and was the most dominant genus at all densities. In addition, *Bacillus* and *Paenibacillus* increased more than twofold in relative abundance with density, while abundances of *Aeromicrobium*, *Devosia*, *Methylotenera*, *Hyphomicrobium*, and *Flavobacterium* all decreased more than twofold when plant density was increased to the highest level.

At all densities, the *T. repens* rhizosphere community was dominated by the genus *Rhizobium*, comprising more than 50% of the community and showing increased relative abundance with increasing plant density. In contrast, an unclassified genus from the family *Burkholderiaceae* (ASV 53), an unclassified *Chloroflexi* (ASV 17), and the genera *Gaiella*, *Pseudarthrobacter* and candidatus *Udaeobacter* decreased more than twofold in relative abundance from the lowest to the highest density (Supplementary Figure S2).

### Diversity and Community Composition in Mixed Cultures

In mixed cultures, the Chao1 richness and Shannon index of the rhizosphere communities of *T. repens* and *C. cyanus* did not change with increasing plant density (Figure 3B). A two-way ANOVA did not reveal any significant differences in richness and Shannon index between the rhizosphere communities of *T. repens* or *C. cyanus* grown in monocultures and in mixed cultures.

For *C. cyanus*, which was a strong competitor in mixed cultures, *Methylotenera*, *Streptomyces* and *Nocardiodes* were the most dominant genera at the lowest plant density (Supplementary Figure S3). None of the three genera showed any consistent trend in relative abundance when plant density was increased. However, some less abundant genera showed changes with plant density. Hence, *Bacillus* increased, whereas *Devosia* and *Flavobacterium* decreased more than twofold in abundance with increasing density.

In the mixed cultures, *Rhizobium* was identified as the dominant genus in the rhizosphere communities of *T. repens*, the weakest competitor in the experiment. The relative abundance of the dominant genus, *Rhizobium*, decreased from 47% to 29% from the lowest to the highest density. In addition, the relative abundance of the genus *Streptomyces* decreased more than threefold with increased plant density. Genera increasing more than twofold in relative abundance with higher plant density included *Bacillus* and *Paenibacillus* (Supplementary Figure S3).

The rhizosphere community of *T. repens* in the mixed cultures had twofold lower relative abundance of *Rhizobium* at the highest density than in the monocultures (Supplementary Figure S3). In contrast, *Streptomyces*, *Bacillus*, and *Paenibacillus* had twofold higher relative abundances at one or more densities in the mixed cultures compared to the monocultures. *C. cyanus* increased its abundance of *Bacillus* (twofold) and *Methylotenera* (up to eightfold) across densities in the mixed cultures compared with the monocultures.

### Stability Versus Dynamics of Bacterial Communities

The above results suggested that the rhizosphere communities of *C. cyanus* had a more stable composition than those of *T. repens* across plant density and community diversity. To enable a more detailed analysis of the relation of the ASVs with the plants, we assigned each ASV to one of three groups for each of the two plant species. The groups were: Core, Unique and Shared, representing ASVs comprising the core microbiome of the plant, ASVs found only in the rhizosphere of one particular plant species and ASVs shared by both plant species, respectively.

**FIGURE 4:**
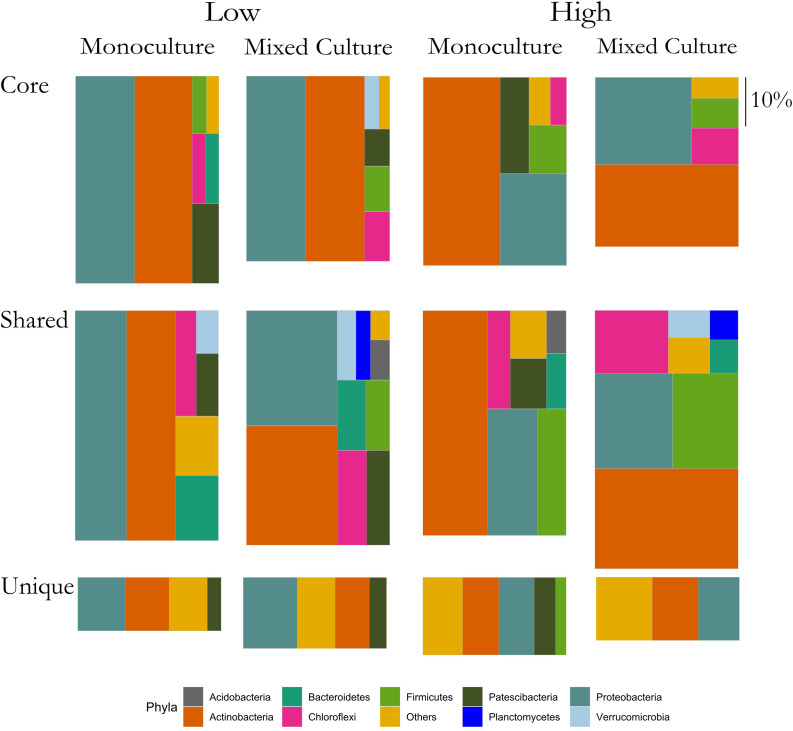
Relative abundances of ASVs classified as Core, Shared, and Unique from rhizosphere bacterial communities of *C. cyanus* grown in monoculture and mixed culture at low and high plant densities. The ASVs are clustered at the phylum level. Phyla that comprise <1% of a group were clustered in “Others.” The size of each rectangle is proportional to the relative abundance of each group. The scale on the right indicates the size of relative proportion of 10% on the vertical scale. The sum of the three boxes in each column is 100%.

**FIGURE 5:**
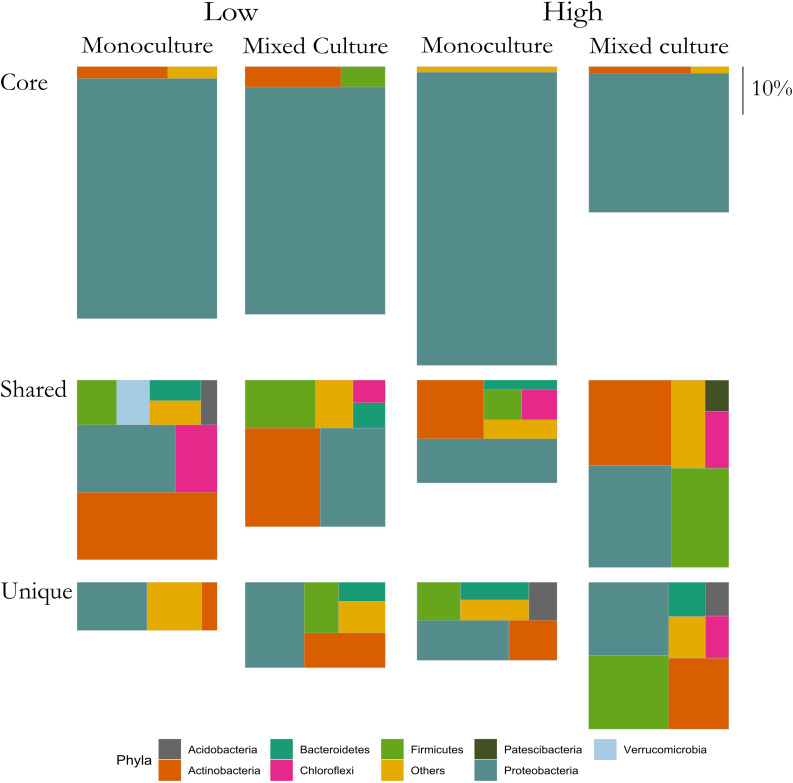
Relative abundances of ASVs classified as Core, Shared and Unique from rhizosphere bacterial communities of *T. repens* grown in monoculture and mixed culture at low and high plant densities. The ASVs are clustered at the phylum level. Phyla that comprise <1% of a group were clustered in “Others.” The size of each rectangle is proportional to the relative abundance of each group. The scale on the right indicates the size of relative proportion of 10% on the vertical scale. The sum of the three boxes in each column is 100%.

**FIGURE 6:**
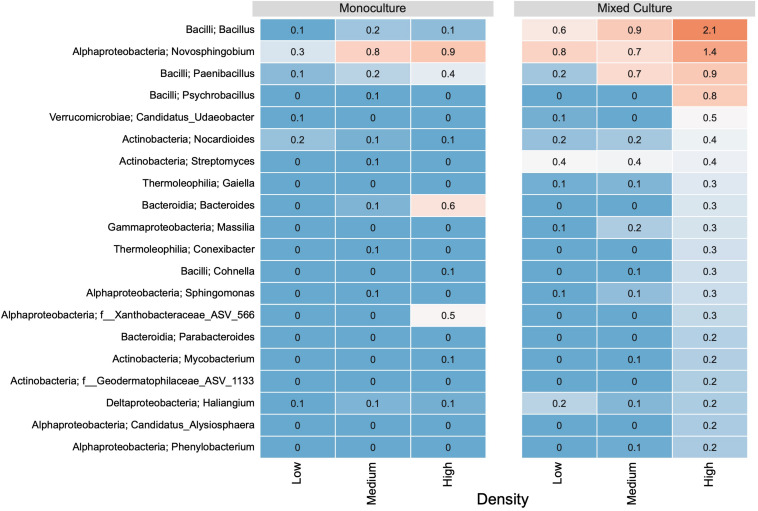
The relative abundance (%) of the 20 most abundant genera based on unique ASVs in bacterial communities from *T. repens* rhizosphere. Plants were grown in monoculture or in mixed culture at low, medium and high plant density. The genera were ranked based on their relative abundance in the highest plant density in the mixed culture. The relative abundances of ASVs are indicated by a color gradient going from blue (low abundance) to red (high abundance).

In the rhizosphere communities of *C. cyanus*, the relative abundances of the Core, Unique and Shared groups were similar among plant densities and across cultivation types (Figure 4). The proportion of Core and Unique ASVs in *T. repens* increased with density in the monocultures (Figure 5). In contrast, the proportion of the Core ASVs decreased substantially from the lowest to the highest density for *T. repens* in the mixed cultures. The decrease of Core ASVs was driven by a reduction of *Rhizobium* ASVs. The Unique and Shared ASVs increased relatively when increasing the density for the mixed cultures. The group of Unique ASVs contributed with the largest increase from 17.8% to 30.6% of the rhizosphere community of *T. repens* in mixed cultures. This increase was driven by increasing relative abundances of ASVs belonging to the genera of *Bacillus*, *Paenibacillus*, *Psychrobacillus*, *Novosphingobium*, and *candidatus* Udaeobacter (Figure 6).

### Impact of Plant Species and Density on *nifH* Gene Abundance

**FIGURE 7:**
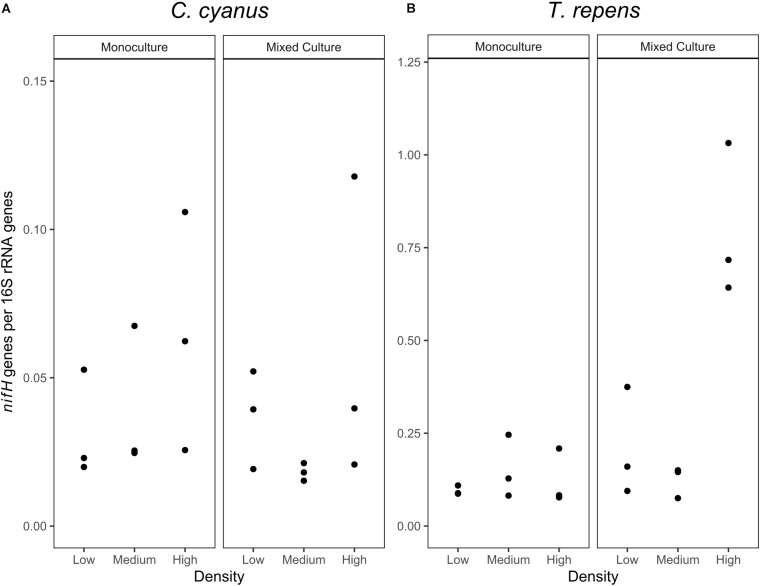
Abundances of *nifH* genes relative to 16S rRNA genes (*n* = 3) in the rhizosphere bacterial communities of *C. cyanus* **(A)** and *T. repens* **(B)** grown in monoculture or mixed culture at low, medium and high plant density.

As the relative abundance of *Rhizobium* decreased in the *T. repens* rhizosphere communities when plants were grown at increasing density in the mixed cultures, we quantified the *nifH* genes to identify the potential for nitrogen fixation of the rhizosphere communities of *T. repens* and *C. cyanus*. The relative abundance of *nifH* genes (*nifH* gene copy number/16S rRNA gene copy number) ranged between 0.08 and 0.24 for *T. repens* in the monocultures (Figure 7) but did not significantly differ between plant densities (ANOVA, *p* = 0.59). In the mixed cultures, the mean relative abundance of *nifH* genes was 0.21 and 0.12 for the low and medium plant densities, respectively, and increased significantly to 0.80 genes per 16S rRNA gene at the highest density (*p* = 0.022 and *p* = 0.0071, respectively). For *C. cyanus* rhizosphere communities, the relative abundance of *nifH* genes was lower than for the *T. repens* rhizosphere communities across densities for both monocultures and mixed cultures (*p* = 0.0044 and *p* = 0.0019, respectively) (Figure 7). The relative abundances of *nifH* genes did not differ among the plant densities in the monocultures or the mixed cultures for *C. cyanus*.

## Discussion

### Plant Competitive Performance

*Centaurea cyanus* was the strongest competitor in our experiment, as its biomass production in mixture was the least reduced as compared to monoculture. On the other hand, *T. repens* was a poor competitor in our experiments. Due to the large number of plants per square meter in the five-species mixture it was not possible to collect and separate the belowground biomass of the plants, so our analysis of plant competition is based on aboveground biomass. Including the belowground biomass would in all likelihood not affect the rankings of competitive performance (Casper and Jackson, 1997). While some plants alter the relationship between above- vs. belowground allocation in response to competition, most changes are allometrically constrained, i.e., they are primarily due the effects of competition on plant size (Müller et al., 2000), and major effects on total biomass will be reflected in aboveground biomass.

### The Rhizosphere Bacterial Communities of Individual Plant Species

In the ordination of bacterial communities in monoculture, those of *T. repens*, the nitrogen-fixing legume and *L. multiflorum*, the grass species, are clearly separated from those of the other three broadleaved species. However, as emphasized by Aleklett et al. (2015), there is currently no general evidence that plant species in the same family level share more similarities in bacterial community composition than more distantly related plants. Across our plant species, the effect of density on the bacterial community was greater in mixture than in monoculture, consistent with our first hypothesis that individual species generally maintain a specific microbiome with increasing density in monoculture.

Our detailed analyses provide the first descriptions of the *C. cyanus* rhizosphere bacterial community composition. The community (at low plant density) is dominated by Alphaproteobacteria and Actinobacteria. At low plant density, the *T. repens* rhizosphere community is dominated by Alphaproteobacteria, Thermolephilia, Bacilli, and Actinobacteria. A previous study based on analysis of clone libraries reported dominance of Gammaproteobacteria (*Pseudomonas*) and Alphaproteobacteria (*Rhizobium*) in bacterial communities associated with *T. repens* (Marilley and Aragno, 1999). A recent community sequencing study on the related species *T. pratense* (red clover) confirmed a very high relative abundance of *Rhizobium* (ca. 70%), and even that OTUs from the Proteobacteria and the Firmicutes were abundant members of the root communities (Hartman et al., 2017).

### Effects of Intraspecific Competition

Recent evidence points to a role of soil microorganisms in plant competition. For example, *Trifolium fucatum* was strongly inhibited by conspecifics in sterile soil, whereas intraspecific competition was diminished when microbes extracted from soil were added to the sterilized soil (Siefert et al., 2018). In contrast, intraspecific competition for *Plantago lanceolata* was increased by the introduction of a microbial inoculum to sterile soil (Hawkins and Crawford, 2018).

As the superior competitor, *C. cyanus* was more affected by intra- than interspecific competition, in terms of its rhizosphere bacterial community as well as biomass production. With increasing plant density in the monocultures, *C. cyanus* rhizosphere communities maintained relatively stable properties of ASVs classified as Core, Shared, and Unique. Nevertheless, increasing the plant density changed the rhizosphere bacterial communities and the plant recruited *Streptomyces*, *Bacillus* and *Paenibacillus*, groups which include plant growth-promoting bacteria (Compant et al., 2019). These changes suggest that the plant recruits mutualistic bacteria during increasing intraspecific competition.

Plants change the composition of their exudates under stress (Gargallo-Garriga et al., 2018). Although we did not investigate root exudates, the decrease of *Methylotenera*, *Hypnomicrobium* and *Flavobacterium* noted for *C. cyanus* with increasing plant density points toward a change in the composition of exudate. This is because these bacterial genera contain known methylotrophs capable of degrading methanol or other 1- carbon compounds (Chistoserdova et al., 2009; Madhaiyan et al., 2010). Methanol is formed during dimethyl esterification of pectin during plant cell growth (Dorokhov et al., 2018). Hence, we hypothesize that reduced plant cell growth results in less methanol being released as exudate, resulting in a decrease in methylotrophs. Along this line, the related species *C. maculosa*, produces higher levels of defense-related metabolites, when grown with conspecific plants, but lower levels of primary, growth-related metabolites than when grown with other neighboring plants (Broz et al., 2010).

For *T. repens* grown in monoculture, the richness and diversity of the rhizosphere communities decreased with density. Accordingly, the communities increased the proportions of core ASVs (the largest fraction was Rhizobiales), as well as of Unique ASVs, but they lost ASVs shared with *C. cyanus.* This suggests that the plant maintains a highly specialized community as intraspecific competition increases. Most legumes such as *T. repens* establish symbiotic relationships with rhizobial strains present in the soil by root nodule formation (Denison and Kiers, 2004). While the relative abundance of *Rhizobium* increased when the plant density increased, this was not accompanied by a higher relative abundance of *nifH* genes involved in nitrogen fixation. Not all *Rhizobium* strains are capable of fixing nitrogen, and the rhizosphere can comprise a vast amount of non-nitrogen-fixing rhizobia (diCenzo et al., 2019). Recent studies have shown that native rhizobia can compete for nodule occupancy with nodulating nitrogen-fixing *Rhizobium* inoculants (diCenzo et al., 2019; Irisarri et al., 2019). Some studies have even suggested that native rhizobia that do not have the appropriate symbiotic efficiency for nitrogen fixation, can be more competitive than nitrogen fixing rhizobia to occupy an important portion of the nodules (Blanco et al., 2010; Yates et al., 2011). In our study, we suggest that in the rhizosphere of *T. repens* competition between nitrogen-fixing rhizobia and non-nitrogen fixing rhizobia might be affected during increasing intraspecific plant competition.

According to our first hypothesis, a stable rhizosphere community is maintained during intraspecific competition. We found considerable changes in community composition, in particular for *T. repens*, so based on our detailed analysis we reject this as a general hypothesis.

### Effects of Interspecific Competition

*Centaurea cyanus* was the superior competitor, and we hypothesized that the *C. cyanus* rhizosphere community would be more stable compared to that of *T. repens*. Indeed, in the mixed cultures, the rhizosphere community of *C. cyanus* showed higher stability at the increasing plant densities than that of *T. repens*. A recent study of competition between the plants *Maytenus senegalensis* and *Lycium intricatum* addressed the effect of interspecific competition on soil microbial communities. That study reported that when the two plant species were grown in mixed cultures, the soil community resembled that found for monocultures of *L. intracatum*, which was the superior competitor (Hortal et al., 2017). We did not observe a comparable effect in the current experiments. Rather, the *T. repens* rhizosphere bacterial communities showed an increase of unique taxa in the mixed culture. However, the results of Hortal et al. (2017) were for communities in the soil adjacent to the plants, while our current study investigates rhizosphere communities that can be assumed to be more profoundly affected by the host plant.

In contrast to the *C. cyanus* communities, the composition of the rhizosphere bacterial communities of *T. repens* changed substantially at increasing plant density. However, the community of *T. repens* did not resemble the community of *C. cyanus* at any of the three tested densities. Rather, we observed a substantial decrease in the relative abundance of the core taxa in the rhizosphere, primarily driven by a decrease of *Rhizobium*. Still, an increase in *nifH* genes was observed indicating a higher potential for nitrogen fixation, despite the decrease in relative abundance of *Rhizobium.* In a maize/faba bean intercropping setup, maize root exudates increased nodulation and nitrogen fixation in faba beans (Li et al., 2009, 2016). While suggesting similar responses in *T. repens* in the mixed culture on the overall nitrogen fixation potential, our study did not investigate whether this was linked to increased nodulation in *T. repens*. Our setup did not allow for determination of accumulated soil nitrogen, but previous results showed no difference in soil nitrogen during intercropping of faba bean and maize.

In response to the reduction in *Rhizobium*, *T. repens* attracted other taxa able to fix nitrogen. The genera *Herbaspirillum*, *Bacillus* and *Paenibacillus* contain species carrying *nifH* genes (Pedrosa et al., 2011; Hong et al., 2012; Rilling et al., 2018); and some *Paenibacillus* have been shown to contain three *nifH* genes (Hong et al., 2012). Interestingly, the decrease in relative abundance of *Rhizobium* was not found in the monocultures for *T. repens* (as discussed above), indicating that a higher diversity of nitrogen fixing taxa results from competition with other plants. The recruitment of these nitrogen-fixing taxa may be explained by plant need for nitrogen, although they are less efficient nitrogen-fixers than *Rhizobium* (Dobbelaere et al., 2003). In addition, newly recruited taxa as *Bacillus*, *Paenibacillus*, and *Streptomyces* harbor several other plant growth promoting traits including phosphorous solubilization, production of phytohormones and antagonism toward plant pathogens (Pieterse et al., 2014; Grady et al., 2016). Consequently, these taxa may offer a broader palette of beneficial functions needed by the *T. repens* plants suffering from interspecific competition.

Taken together, the results for the mixed communities support the first part of our second hypothesis, that a dominant species maintains it microbiome under increasing interspecific competition, while that of a poor competitor changes. But the results do not support the second part of the hypothesis, that the microbiome of the poor competitor becomes more similar to that of the strong competitor.

## Conclusion

Competition among plants has major effects on the plants’ rhizosphere bacterial communities. Across the plant species in our experiment, the effects of interspecific competition were different and larger than those of intraspecific competition. For the dominant competitor, *C. cyanus*, increasing intra- as well as interspecific competition lead to comparable changes in the rhizosphere bacterial communities; while for the weakest competitor, *T. repens*, the effects of interspecific competition were particularly conspicuous. Our results represent a starting point for further studies addressing the interactions between plant competition and the rhizosphere microbiome.

## Data Availability Statement

The raw sequences were deposited in NCBI’s Sequence Read Archive under BioProject PRJNA574065.

## Author Contributions

AC, JW, ON, and MN designed the experiments. AC carried out the field work. FB analyzed the sequencing data. AC and AG-L carried out the laboratory experiments. All authors contributed to the writing and editing of the manuscript and approved the final version.

## Conflict of Interest

The authors declare that the research was conducted in the absence of any commercial or financial relationships that could be construed as a potential conflict of interest.
