# Supplementary material for: Effects of Intra- and Interspecific Plant Density on Rhizosphere Bacterial Communities
Source: Front Microbiol. 2020 May 26;11:1045. doi: 10.3389/fmicb.2020.01045 (PMC7264394; doi:10.3389/fmicb.2020.01045)
Supplement: Supplementary file 1 [file Data_Sheet_1.docx]

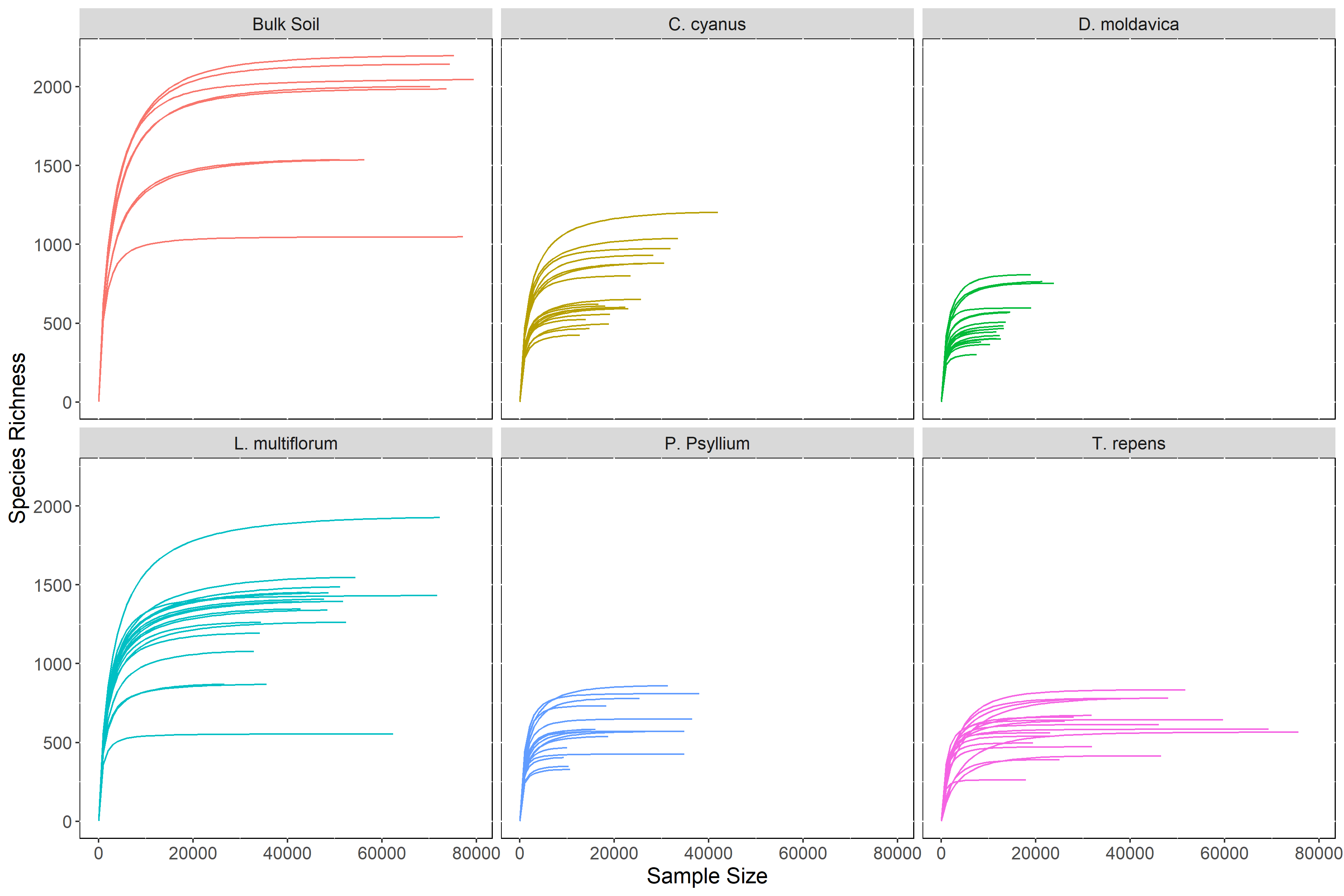


**Figure S1.** Rarefaction curves of samples from the rhizosphere of the five plant species and from the bulk soil.

**
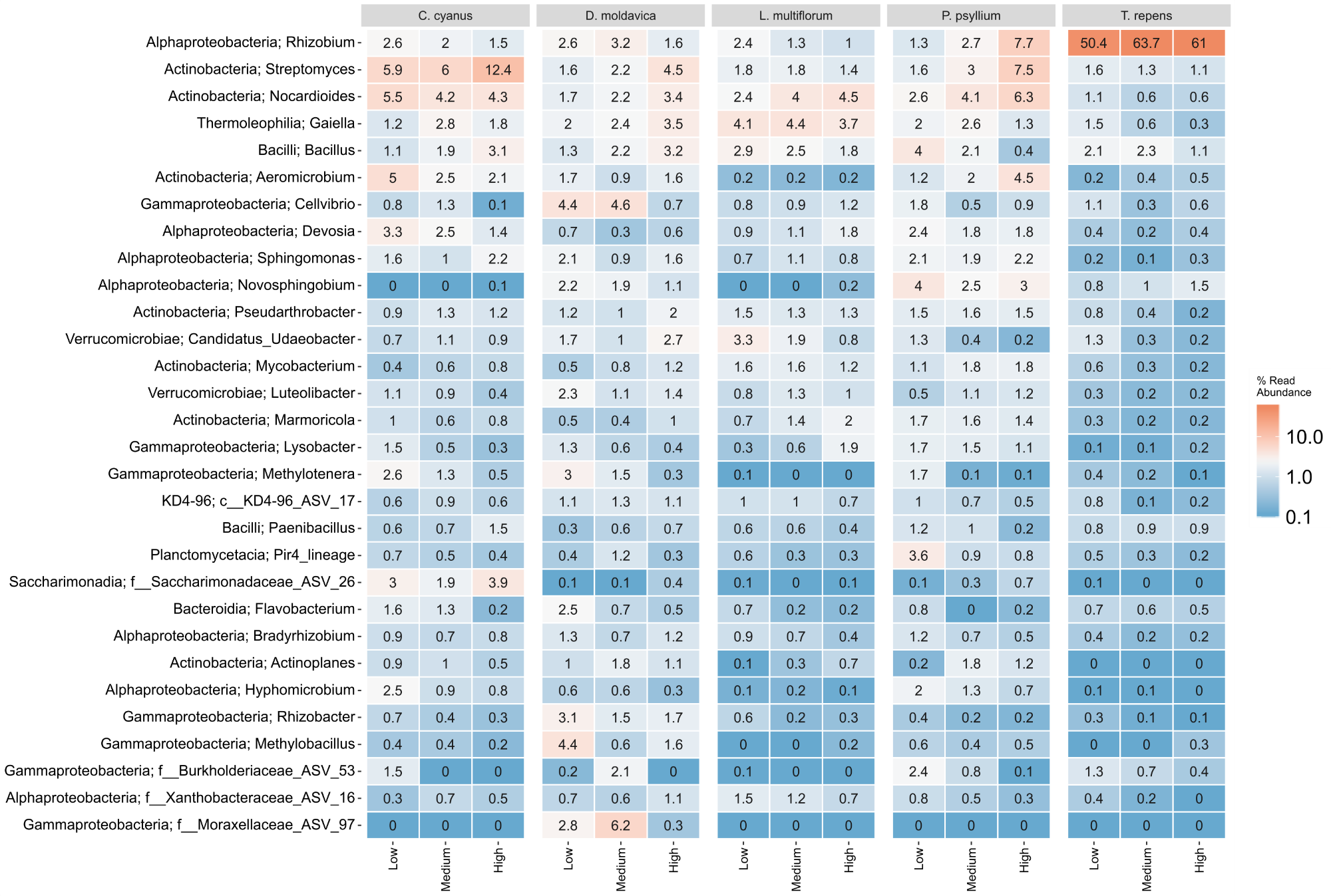
**

**Figure S2.** Heatmap of the 30 most abundant genera across 5 plant species and densities in mono culture (Low, Medium and High) (n = 3).


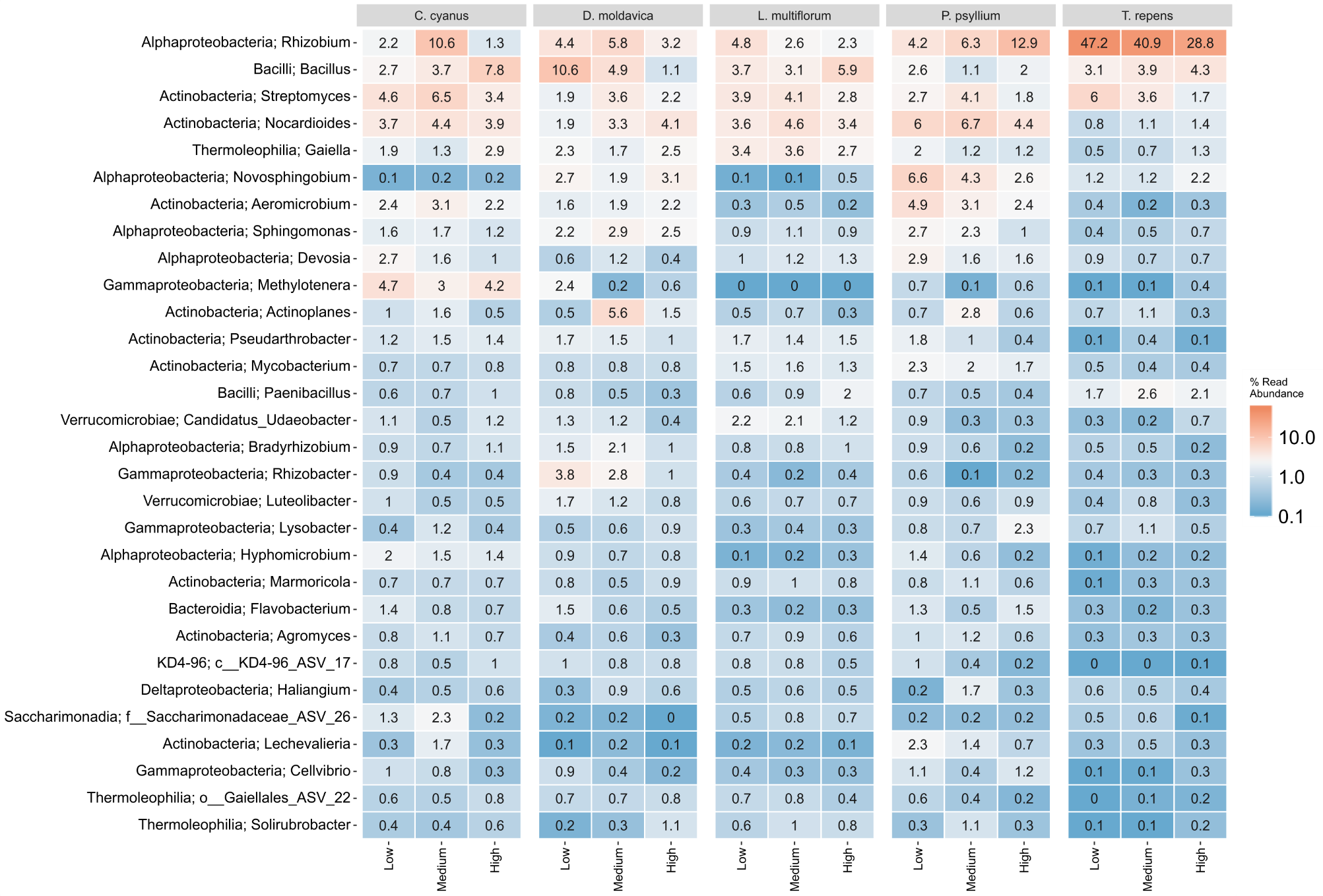


**Figure S3.** Heatmap of the 30 most abundant genera across 5 plant species and densities in mixed culture (Low, Medium and High) (n = 3).

**
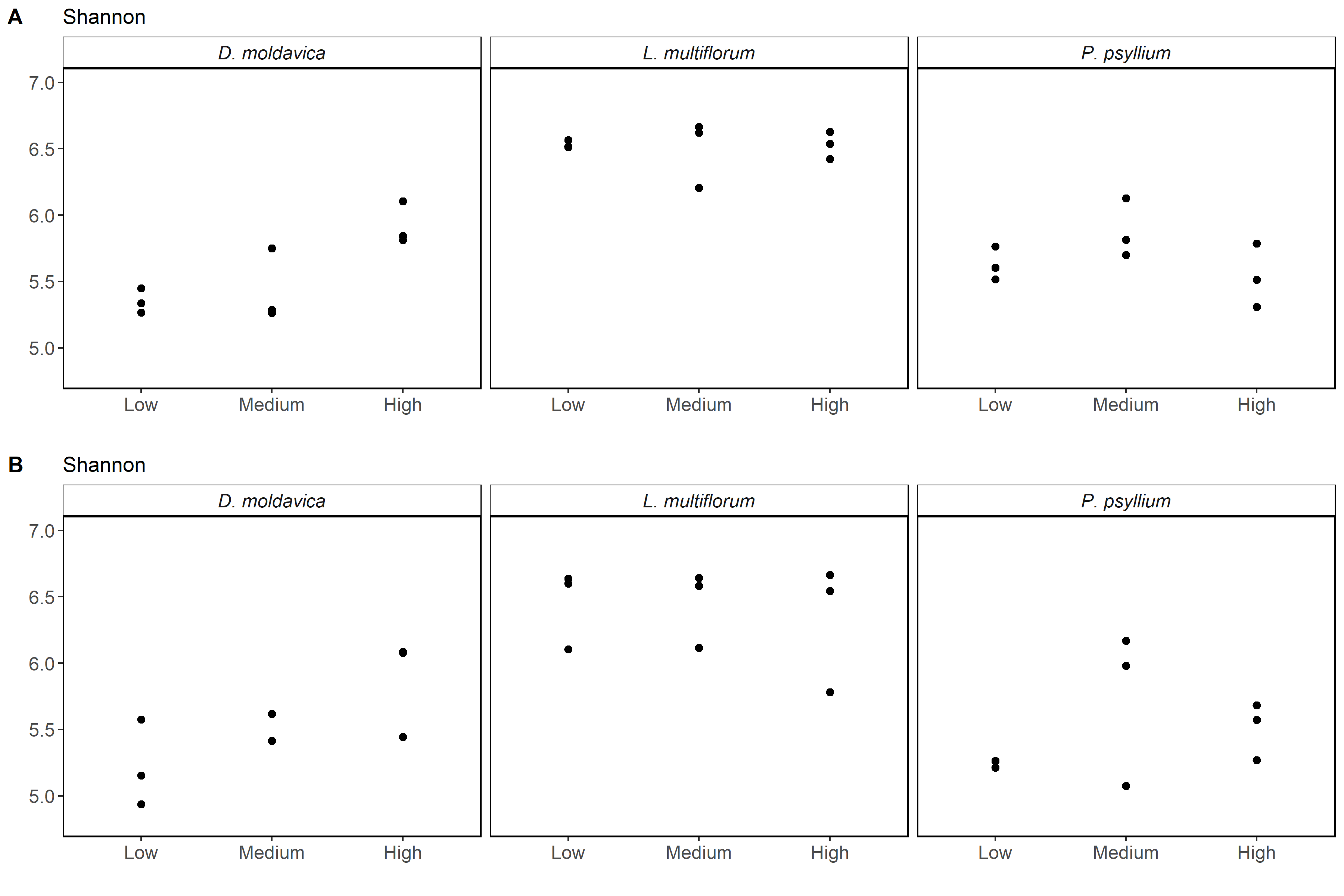
**

**Figure S4**. Shannon index for rhizosphere bacterial communities of *D. moldavica*, *L. multiflorum* and *P. psylliym* grown in monoculture (A) or in mixed culture (B) at three plant densities (low, medium and high) (n = 3).

**
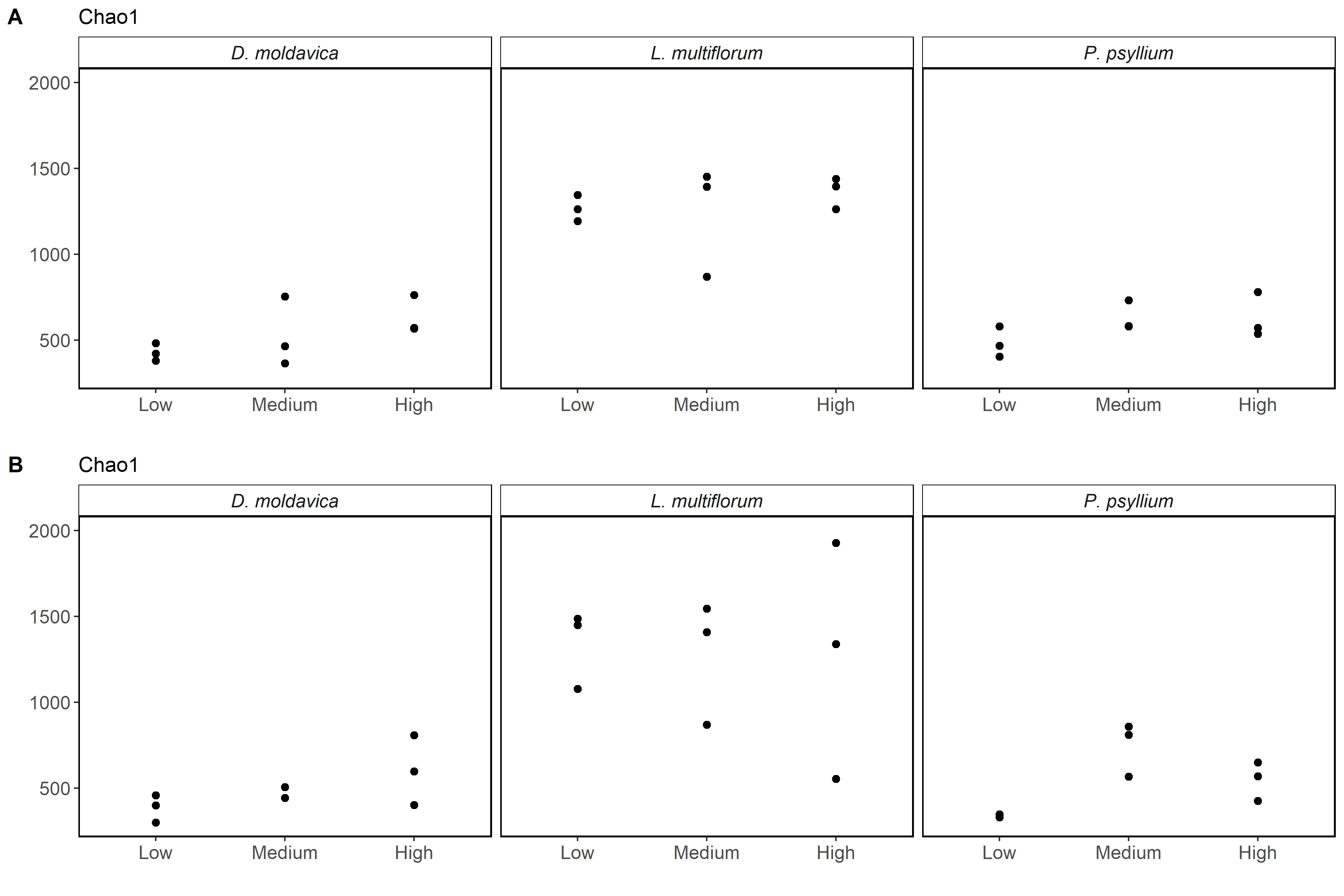
**

**Figure S5**. Chao1 richness for rhizosphere bacterial communities of *D. moldavica*, *L. multiflorum* and *P. psylliym* grown in monoculture (A) or in mixed culture (B) at three plant densities (low, medium and high) (n = 3).
